# Predictive Factors of Clinical Response of Infliximab Therapy in Active Nonradiographic Axial Spondyloarthritis Patients

**DOI:** 10.1155/2015/876040

**Published:** 2015-07-26

**Authors:** Zhiming Lin, Zetao Liao, Jianlin Huang, Maixing Ai, Yunfeng Pan, Henglian Wu, Jun Lu, Shuangyan Cao, Li Li, Qiujing Wei, Deshen Tang, Yanlin Wei, Tianwang Li, Yuqiong Wu, Manlong Xu, Qiuxia Li, Ou Jin, Buyun Yu, Jieruo Gu

**Affiliations:** ^1^Department of Rheumatology, The Third Affiliated Hospital of Sun Yat-Sen University, Tianhe Road No. 600, Guangzhou 510630, China; ^2^Medical Affairs, Xian-Janssen Pharmaceutical Ltd, Beijing, China; ^3^Department of Rheumatology, Dongguan People's Hospital, Guangdong, China; ^4^Department of Rheumatology, The Affiliated Hospital of Guangdong Medical College, Guangzhou, China

## Abstract

*Objectives.* To evaluate the efficiency and the predictive factors of clinical response of infliximab in active nonradiographic axial spondyloarthritis patients. *Methods.* Active nonradiographic patients fulfilling ESSG criteria for SpA but not fulfilling modified New York criteria were included. All patients received infliximab treatment for 24 weeks. The primary endpoint was ASAS20 response at weeks 12 and 24. The abilities of baseline parameters and response at week 2 to predict ASAS20 response at weeks 12 and 24 were assessed using ROC curve and logistic regression analysis, respectively. *Results.* Of 70 axial SpA patients included, the proportions of patients achieving an ASAS20 response at weeks 2, 6, 12, and 24 were 85.7%, 88.6%, 87.1%, and 84.3%, respectively. Baseline MRI sacroiliitis score (AUC = 0.791; *P* = 0.005), CRP (AUC = 0.75; *P* = 0.017), and ASDAS (AUC = 0.778, *P* = 0.007) significantly predicted ASAS20 response at week 12. However, only ASDAS (AUC = 0.696, *P* = 0.040) significantly predicted ASAS20 response at week 24. Achievement of ASAS20 response after the first infliximab infusion was a significant predictor of subsequent ASAS20 response at weeks 12 and 24 (wald *χ* ^2^ = 6.87, *P* = 0.009, and wald *χ* ^2^ = 5.171, *P* = 0.023). *Conclusions.* Infliximab shows efficiency in active nonradiographic axial spondyloarthritis patients. ASDAS score and first-dose response could help predicting clinical efficacy of infliximab therapy in these patients.

## 1. Introduction

The spondyloarthritis (SpA) is a group of related inflammatory diseases including ankylosing spondylitis (AS), reactive arthritis, psoriatic arthritis, inflammatory bowel disease-associated arthritis, juvenile spondylitis, and undifferentiated spondylitis [1]. The occurrence of SpA is common in many countries; in China the pooled prevalence of SpA from civilian surveys is 0.93%, and for AS is 0.24% [2].

Axial SpAs comprise AS and nonradiographic axial SpA. A previous study showed that the frequency of HLA-B27 positivity, inflammatory back pain, arthritis, enthesitis, uveitis, and levels of disease activity are highly comparable between patients with these two types of diseases, thus suggesting that these two entities are part of the same disease [3]. Thus, the axial SpA patients without radiographic change would partly include the early stage of AS patients.

Following preclinical studies identified the key role of TNF*α* in the immune-mediated inflammatory response observed in AS [4], and anti-TNF*α* agents have been evaluated and approved as for treatment of AS [5]. While numerous studies have assessed anti-TNF*α* agents in patients with established disease per the modified New York criteria, that is, structural changes in the sacroiliac joint are visible on X-ray, few studies have been conducted to ascertain the benefits to treat patients in the early stages of AS or nonradiographic axial SpA [6, 7].

In addition, anti-TNF-*α* agents can be effective in approximately 60%–80% of AS patients [5]; however, the cost of such therapy must be considered in assessing available treatment options, especially in China. Identifying baseline disease characteristics with strong ability to predict efficacy would be quite important in lessening the economic burden of effective treatment for both the patients and the healthcare system in general.

As such, we conducted the current study to evaluate the efficacy of infliximab (REMICADE, Centocor Ortho Biotech Inc, Horsham, PA), an anti-TNF-*α* agent approved for the treatment of active nonradiographic axial spondyloarthritis patients, in patients to assess (1) the ability of baseline disease characteristics and initial clinical response at week 2 to predict the clinical efficacy of infliximab at week 12 and (2) the clinical efficacy of infliximab in active nonradiographic axial spondyloarthritis patients through week 24.

## 2. Patients and Methods

### 2.1. Patients

All patients were recruited by the Department of Rheumatology of the Third Affiliated Hospital of Sun Yat-Sen University from June 2007 to December 2008. In this study, all patients were required to meet the European Spondyloarthropathy Study Group (ESSG) criteria for SpA [1] but could not meet the modified New York criteria for AS [8]. Specifically, patients could not have displayed X-ray evidence of structural changes in the sacroiliac joint (bilateral grade 2 or unilateral grade 3). All axial SpA patients were also required to have less than two-year disease duration and inflammatory back pain (Calin's criteria). In addition, active inflammatory lesions in the sacroiliac joints by MRI were required to be detected in all patients. All patients were required to have a Bath Ankylosing Spondylitis Disease Activity Index (BASDAI) score ≥30 mm (based on a visual analog scale (VAS) ranging from 0 to 100 mm) [9] and to have been receiving stable doses (for at least 4 weeks before baseline) of a single nonsteroidal anti-inflammatory drug (NSAID), if an NSAID was being used; no additional AS therapy was permitted during the 24 weeks preceding baseline.

In addition, if patients with or without peripheral symptoms met the above inclusion criteria, they would be included in our study. Our clinicaltrials.gov identifier number is NCT00936143.

This study was conducted at a single center in China. The independent ethics committee at the study site reviewed and approved the study protocol. Patients provided written informed consent before any study-related procedures were performed.

### 2.2. Patient Treatment and Evaluations

Patients received infliximab 5 mg/kg by intravenous infusion at weeks 0, 2, 6, 12, 18, and 24. Infliximab is a recombinant IgG1-*κ* human-murine chimeric monoclonal antibody that specifically binds to both soluble and membrane-bound forms of TNF*α*. Infliximab is supplied as a sterile, white, lyophilized powder in single-use 20 mL vials.

The following clinical and laboratory determinations were made at weeks 0, 2, 6, 12, 18, and 24: ASDAS, BASDAI, Bath Ankylosing Spondylitis Functional Index (BASFI) [10], erythrocyte sedimentation rate (ESR), and serum C-reaction protein (CRP) concentration. The BASDAI score, which ranges from 0 to 100 mm, is a combined assessment of fatigue, spinal pain, joint pain, enthesitis, and morning stiffness [9]. The BASFI score, which also ranges from 0 to 100 mm, includes 8 questions relating to the patient's function and 2 questions relating to a patient's ability to cope with everyday life [10].

The SPARCC MRI scoring method for spine bone edema was assessed on sagittal slices in two segments (C1-T10 and T10-S2), and active nonradiographic axial spondyloarthritis patients also had X-ray and MRI evaluations of the sacroiliac joints (SIJ) and lower spine (T10-S2 vertebrae) at baseline and week 24 in our study. Active inflammatory lesions of the SIJ and lower spine (T10-S2 vertebrae) were scored according to the SPARCC score system [11–13]. The SPARCC score system for active inflammatory lesions relies on the use of a T2-weighted sequence that incorporates suppression of normal marrow fat signal. All scores are based on abnormal increased signals on the STIR sequence representing increased concentration of “free water” otherwise referred to as “bone marrow edema.” The scoring method described below assumes that images have been acquired according to our MRI acquisition protocol described in this website (available at http://www.arthritisdoctor.ca/) [11–13].

We also determined the proportions of patients achieving at least 20% improvement in the Assessment of Ankylosing Spondylitis (ASAS) International Working Group response criteria [14]. An ASAS20 response was defined as at least 20% improvement (and an absolute improvement from baseline of at least 10 units, on a scale of 0–100 mm) in at least 3 of the following 4 assessment domains: patient's global assessment, spinal pain, physical function according to the BASFI, and morning stiffness (the average of the last 2 questions of the BASDAI). These ASAS20 responders also must not have had deterioration from baseline (defined as a worsening of at least 20% and an absolute worsening of at least 10 units, on a scale of 0–100 mm) in the potential remaining assessment domain. ASAS40 criteria (at least 40% improvement and 20 units of absolute change in 3 of 4 domains, using the same domains as the ASAS response criteria, without any worsening in the fourth domain) and ASAS partial remission (defined as a value of 20 on a 0–100 mm scale in each of the 4 ASAS20 domains (patient's global assessment, pain, function, and inflammation) were also used for assessing the efficacy [15]. Clinical response was also assessed using the recently developed Ankylosing Spondylitis Disease Activity Score (ASDAS) which was developed specifically for the patients with AS [16, 17].

### 2.3. Statistical Methods

For analyzing the efficacy of treatment during the 24 weeks, we considered those failing to complete 24 weeks' treatment as treatment failure and conducted analysis by nonresponder imputation.

We assessed the ability of baseline indicators of inflammation and disease severity, that is, ESR, CRP concentration, and ASDAS, BASDAI, BASFI, and MRI sacroiliitis score, to predict attainment of an ASAS20 response at weeks 12 and 24 using Receiver Operating Characteristic (ROC) curve methodology. *P* value less than 0.05 was considered statistically significant. We also assessed the ability of ASAS20 response at week 2 to predict the attainment of such a response at week 12 and week 24 using logistic regression analysis.

## 3. Results

### 3.1. Patient Disposition and Baseline Characteristics

Seventy active nonradiographic axial spondyloarthritis patients were enrolled at a single study center. All patients enrolled completed the week-12 visit, and 61 patients completed the week-24 visit. Nine patients discontinued infliximab treatment at week 12: 3 patients due to adverse events (2 with allergies, 1 with tuberculosis) and 6 patients due to an unsatisfactory therapeutic response.

In this study, 82.9% of patients were men, 90.0% were HLA-B27-positive, and 18.6% had a family history of AS. The disease duration of active nonradiographic axial spondyloarthritis patients was 1.41 ± 0.57 years. MRI scores for the sacroiliac joint and lower spine (T10-S2 vertebrae) were 20.40 ± 10.44 and 1.86 ± 3.85, respectively (Table 1). 100% and 90% of patients fulfilled the new ASAS axial spondyloarthritis classification criteria set 1 and set 2, respectively [18]. In other words, all patients that were included were nonradiographic axial spondyloarthritis patients.

### 3.2. Infliximab Clinical Efficacy in Active Nonradiographic Axial Spondyloarthritis Patients

Efficacy endpoints were assessed at week 12 and week 24.

#### 3.2.1. Week 12

Among the 70 enrolled active nonradiographic axial spondyloarthritis patients, similar proportions of patients achieved an ASAS20 response at weeks 2, 6, and 12 (85.7%, 88.6%, and 87.1%, resp., *P* = NS). Proportions of patients who achieved an ASAS40 response at weeks 2, 6, and 12 were 61.43%, 62.86%, and 67.14%, respectively (*P* = NS). Proportions of patients who achieved an ASAS partial remission at weeks 2, 6, and 12 were 57.14%, 58.58%, and 60.0%, respectively (*P* = NS). Proportions of patients who achieved BASDAI50 response at weeks 2, 6, and 12 were 67.14%, 71.43%, and 74.29%, respectively (*P* = NS). ASDAS, which decreased from 3.02 ± 1.25 at baseline (high disease activity) to 0.83 ± 0.63 at week 2, 0.75 ± 0.54 at week 6, and 0.76 ± 0.68 at week 12, suggested clinically significant improvement in these patients (*P* = 0.000) and continuing through week 12. Significant improvements were also observed in the BASDAI score, BASFI score, CRP concentration, and ESR at the beginning of week 2, compared to baseline (*P* = 0.000) and continued to week 12.

#### 3.2.2. Week 24

For analyzing the efficacy of treatment during the 24 weeks, we considered nine patients who failed to complete 24 weeks' treatment as treatment failure and conducted analysis by nonresponder imputation.

At week 24, 84.3% of patients achieved an ASAS20 response. Besides, the proportions of the patients achieving an ASAS40 response, ASAS partial remission, and BASDAI50 response were 64.17%, 61.43%, and 68.57%, respectively.

### 3.3. Ability of Baseline Disease Characteristics and Early Clinical Response to Predict Infliximab Clinical Efficacy in Active Nonradiographic Axial Spondyloarthritis Patients

#### 3.3.1. Week 12

**Figure 1:**
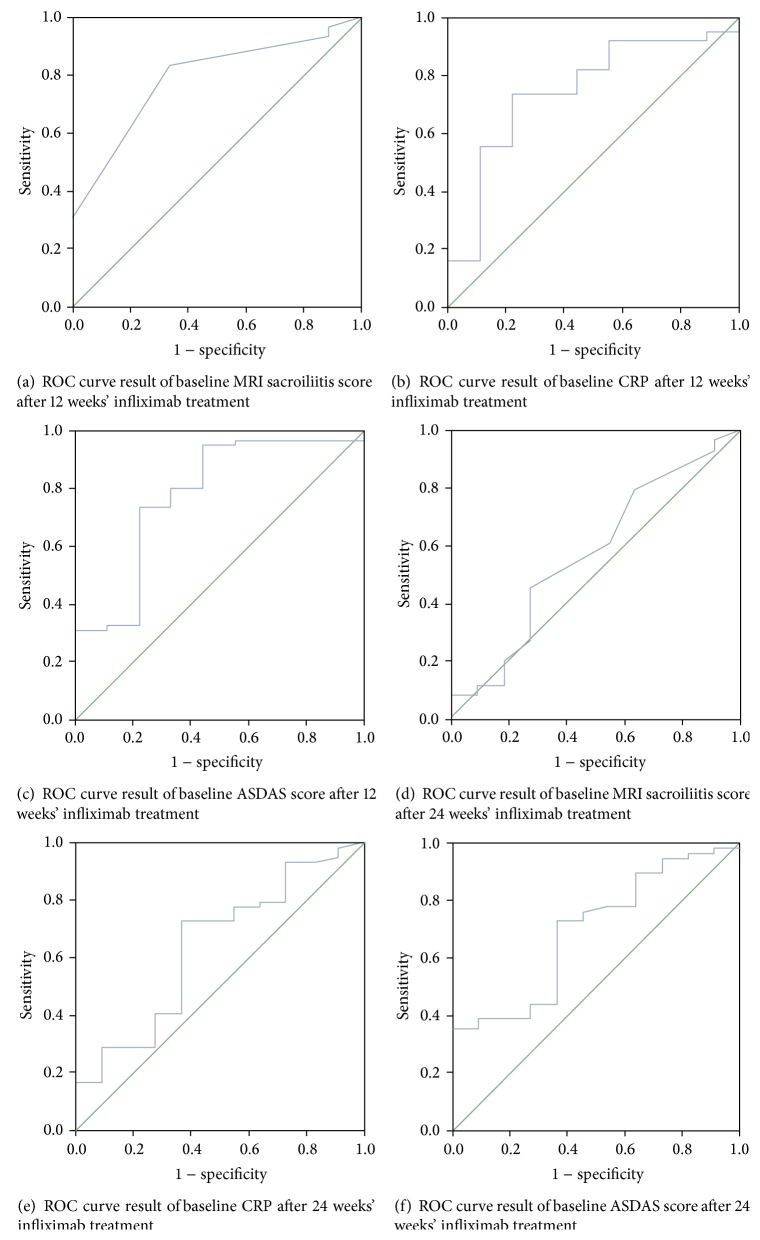
Ability of baseline sacroiliac joint magnetic resonance imaging (MRI) score (Panel (a)), serum C-reactive protein (CRP) concentration (Panel (b)), and Ankylosing Spondylitis Disease Activity Score (ASDAS) (Panel (c)) to predict at least 20% improvement in the Assessment of Ankylosing Spondylitis International Working Group response criteria (ASAS20) from baseline to week 12, as determined by area under the Receiver Operating Characteristics (ROC) curve. The same analysis results of sacroiliac joint magnetic resonance imaging (MRI) score, serum C-reactive protein (CRP) concentration (Panel (b)), and Ankylosing Spondylitis Disease Activity Score (ASDAS) at week 24 were shown in Panel (d), Panel (e), and Panel (f).

Results of ROC curve analysis indicated that the baseline MRI sacroiliitis score was a significant predictor of achievement of an ASAS20 response at week 12 after 3 infliximab infusions (AUC = 0.791; *P* = 0.005) (Figure 1(a)). Baseline CRP (AUC = 0.75; *P* = 0.017) (Figure 1(b)) and baseline ASDAS (AUC = 0.778, *P* = 0.007) (Figure 1(c)) were also significant predictors of ASAS20 response at week 12. The other baseline parameters assessed, including BASDAI (AUC = 0.556; *P* = 0.593), BASFI (AUC = 0.644; *P* = 0.166), and ESR (AUC = 0.618; *P* = 0.254), were not significant predictors of ASAS20 response at week 12.

Considering CRP, ASDAS, SIJ MRI score at baseline, and age as corrected factors, we analyzed ASAS20 response at week 2 by stepwise regression method (logistic regression) after 12 weeks' treatment and 24 weeks' treatment.

Finally, considering CRP, ASDAS, SIJ MRI score at baseline, and age as corrected factors, we analyzed ASAS20 response at week 2 by stepwise regression method (logistic regression) after 12 weeks' treatment. The result showed that an ASAS20 response at week 2 was a significant predictor of subsequent clinical response in the same patient at week 12 (wald  *χ* ^2^ = 6.87, OR = 12.077, *P* = 0.009) (Table 2).

#### 3.3.2. Week 24

As Figure 1 shows, ROC curve analysis indicated that baseline ASDAS (AUC = 0.696, *P* = 0.040) was also significant predictors of ASAS20 response at week 24. However, the baseline MRI sacroiliitis score, CRP, BASDAI, BASFI, and ESR could not predict the achievement of an ASAS20 response at week 24 after 6 infliximab infusions (MRI sacroiliitis score: AUC = 0.575; *P* = 0.434; CRP: AUC = 0.641; *P* = 0.140; BASDAI: AUC = 0.649; *P* = 0.118; BASFI: AUC = 0.625; *P* = 0.191; ESR: AUC = 0.558; *P* = 0.545) (Figures 1(d), 1(e), and 1(f)).

Likewise, considering CRP, ASDAS, SIJ MRI score at baseline, and age as corrected factors, we analyzed ASAS20 response at week 2 by stepwise regression method (logistic regression) after 24 weeks' treatment. The result showed that an ASAS20 response at week 2 was a significant predictor of subsequent clinical response in the same patient at week 24 (wald  *χ* ^2^ = 5.171, OR = 6.764, *P* = 0.023) (Table 2).

## 4. Discussion

Few studies reported the efficacy for active axial SpA with TNF-*α* blocker treatment. Sieper et al. reported that adalimumab has a positive benefit-risk profile in active nr-axSpA patients with inadequate response to NSAIDs [7]. However, it is rarely reported about the efficacy for active axial nr-SpA with infliximab treatment, and our results suggested infliximab may be an effective treatment for active axial nr-SpA. The results of our study confirmed that TNF-*α* blocker has a significant efficacy for early axial SpA patients, and, moreover, we detected four indexes for predicting clinical efficacy with infliximab therapy in active axial SpA patients, which included ASDAS score, MRI sacroiliitis score, CRP, and first dose response.

So far high CRP, high MRI spine score, and lower BASFI were reported to be predictors of major clinical responses to anti-TNF therapy in patients with active AS patients [19–21]. However, we firstly report that ASDAS score and first doses response are the predictors of clinical response to infliximab therapy in active axial SpA patients.

As is known to us all, ASDAS score is a good index for assessing the disease activity, but it is unknown whether it predicts the clinical response in active axial SpA patients [22]. This study firstly demonstrates the ability of the baseline ASDAS to predict the efficacy for active nonradiographic axial spondyloarthritis patients after 12 weeks' and 24 weeks' infliximab therapy. ASDAS is a highly discriminatory instrument for assessing disease activity in AS that includes an objective inflammatory marker (serum CRP concentration) and subjective assessments of disease activity. Based on these, the ASDAS score provides information regarding the overall state of inflammation in AS patients [16, 17, 22]. The results of this study showed that BASDAI at baseline could not predict the efficacy of infliximab therapy but ASDAS could. As we knew, the difference between ASDAS and BASDAI was that ASDAS included an objective inflammatory marker (serum CRP concentration). It may be a possible reason why ASDAS at baseline can predict the efficacy of infliximab therapy but BASDAI cannot. In consistence with this supposition, our result suggested that baseline serum CRP concentration was a significant predictor of clinical response to infliximab therapy at week 12, which was consistent with findings of Luc and colleagues [19]. Luc's study showed that baseline serum CRP concentration was the only factor out of several evaluated that predicted AS patient continuation of anti-TNF therapy, as opposed to discontinuing such therapy because of lack of efficacy [19].

Besides, ASDAS, MRI sacroiliitis score were another significant predictor of clinical response to infliximab therapy at week 12 in our study. MRI has emerged as a key tool in diagnosing axial SpA [23] based on its ability to detect the bone marrow edema that precedes structural changes in the spine, but the research on the predictive ability of MRI scores in AS and axial SpA is limited. Rudwaleit and colleagues reported that the MRI spine score was predictive of a major clinical response to anti-TNF therapy in patients with active AS [20]. In their study, Rudwaleit and colleagues defined a major clinical response to anti-TNF therapy as achievement of a BASDAI50 (at least 50% improvement in the BASDAI) response. In addition, their results indicated that inflammation of the spine, but not the sacroiliac joint, as detected by MRI significantly predicted clinical efficacy of anti-TNF therapy at week 12 [19]. Nevertheless, the MRI sacroiliitis score was a significant predictor of achievement of an ASAS20 response after 3 infliximab infusions at week 12 in our study. As a possible explanation for these differences between study results, we note that patients in the Rudwaleit study had AS for an average of 14 years, compared with 1.4 years in the our study. And during the initial stages of axial SpA, inflammation in the sacroiliac joints is typically evident within the first 2 years of disease, even when spinal mobility remains normal. Only 19 patients (27.14%) were detected in having inflammation of the spine by MRI in our study. Taken together, these MRI findings suggest that the sacroiliac joint is a more important location for MRI assessment in active nonradiographic axial spondyloarthritis patients, while the spine should be the focus of MRI assessments in AS patients with much more established disease. In addition, results of these 2 studies suggest that MRI scores can help predict subsequent clinical response to anti-TNF therapy in axial SpA patients. And sacroiliac joint MRI plays a more important role in active nonradiographic axial spondyloarthritis patients.

In addition, the ability of active nonradiographic axial spondyloarthritis patients to achieve an ASAS20 response 2 weeks following an initial infliximab infusion was a significant predictor of the same patient's ability to also achieve this response at week 12 (i.e., following 3 infliximab infusions). It was an interesting and important finding. As we know, although infliximab efficacy has been documented in AS [21, 24–26], the cost of infliximab therapy can deter its use in the clinical setting. Having the ability to predict which patients might benefit the most from infliximab therapy could help guiding the clinician in appropriately allocating limited healthcare resources. Our results could be particularly valuable to the physicians when deciding whether to start or continue with a patient's infliximab therapy.

Moreover, previous studies suggested that lower BASFI scores were predictors of a major clinical response to TNF blockers in active AS [27]. However, in our study, BASFI at baseline cannot predict the efficacy in active nonradiographic axial spondyloarthritis patients after infliximab treatment. As the result shown, mean BASFI which only was 23.41 ± 19.46 mm in our study was significantly less than previous study (reach 54 mm) [27]. It may be the reason why our result was different from previous study. Therefore, these data need to be confirmed in further studies.

However, the baseline MRI sacroiliitis score and CRP could not predict the achievement of an ASAS20 response at week 24 after 6 infliximab infusions, although they were considered as predictors of clinical response at week 12 after 3 infliximab infusions in our study. But ASDAS still showed its superiority of predicting the clinical response after infliximab treatment at week 24. Taking the results above into consideration, ASDAS score may be a more sensitive indicator than other indicators for predicting the clinical response in axial SpA patients with infliximab treatment.

In conclusion, infliximab has a high efficiency in active nonradiographic axial spondyloarthritis patients. ASDAS score and first dose response could effectively help predict clinical efficacy with infliximab therapy in these patients.

## Figures and Tables

**Table 1:** Baseline patient characteristics.

	Axial SpA with less than two-year disease duration (*n* = 70)
Age (years)	21.00 ± 7.05
Sex	
Male	58 (82.9%)
Female	12 (17.1%)
Family history of AS	13 (18.6%)
HLA-B27(+)	63 (90.0%)
Peripheral symptoms (arthritis or enthesitis)	17 (24.3%)
Disease duration (years)	1.41 ± 0.57
BASDAI (0–100 mm VAS)	46.86 ± 10.48
BASFI (0–100 mm VAS)	23.41 ± 19.46
BAS-G (0–100 mm VAS)	49.95 ± 19.16
ASDAS	3.02 ± 1.25
MRI score	
Sacroiliac joint	20.40 ± 10.44
Lower spine (T10-S2)	1.86 ± 3.85
Modified Schober test (cm)	4.63 ± 1.69
CRP (mg/L)	31.43 ± 38.33
ESR (mm/hr)	34.75 ± 30.48

Data reported are mean (standard deviation) or number (%) of patients.

BASDAI: Bath Ankylosing Spondylitis Disease Activity Index, BASFI: Bath Ankylosing Spondylitis Functional Index, BAS-G: Bath Ankylosing Spondylitis Global assessment, CRP: C-reactive protein, ESR: erythrocyte sedimentation rate, HLA: human leukocyte antigen, MRI: magnetic resonance imaging, and VAS: visual analog scale.

**Table 2:** Considering CRP, ASDAS, SIJ MRI score at baseline, and age as corrected factors to analyze ASAS20 response at week 2 by stepwise regression method (logistic regression) after 12 weeks' treatment and 24 weeks' treatment.

	After 12 weeks' treatment	After 24 weeks' treatment
Wald *χ* ^2^	6.870	5.171
*P* value	0.009	0.023
OR	12.077	6.764
